# Surface Plasmon Resonance Based Measurement of the Dielectric Function of a Thin Metal Film

**DOI:** 10.3390/s18113693

**Published:** 2018-10-30

**Authors:** Radek Chlebus, Jakub Chylek, Dalibor Ciprian, Petr Hlubina

**Affiliations:** Department of Physics, Technical University Ostrava, 17. listopadu 15, 708 33 Ostrava-Poruba, Czech Republic; radek.chlebus@vsb.cz (R.C.); jakub.chylek.st@vsb.cz (J.C.); dalibor.ciprian@vsb.cz (D.C.)

**Keywords:** surface plasmon resonance, Kretschmann configuration, reflectance ratio, resonance wavelength, dielectric function, Drude–Lorentz model, gold

## Abstract

A spectral method based on surface plasmon resonance (SPR) in air is used to measure the dielectric function of a thin metal film. The method utilizes the spectral dependence of the ratio of the reflectances of *p*- and *s*-polarized waves measured in the Kretschmann configuration at different angles of incidence. By processing these dependences in the vicinity of a dip, or equivalently near the resonance wavelength, and using the dispersion characteristics of a metal film according to a proposed physical model, the real and imaginary parts of the dielectric function of the metal can be determined. The corresponding dielectric function of the metal is obtained by a least squares method for such a thickness minimizing the difference between the measured and theoretical dependence of the resonance wavelength on the the angle of incidence. The feasibility of the method is demonstrated in measuring the dielectric function of a gold film of an SPR structure comprising an SF10 glass prism and a gold coated SF10 slide with an adhesion film of chromium. The dielectric function according to the Drude–Lorentz model with two additional Lorentzian terms was determined in a wavelength range from 534 to 908 nm, and the results show that the gold film is composed of homogenous and rough layers with thicknesses 42.8 nm and 2.0 nm, respectively. This method is particularly useful in measuring the thickness and dielectric function of a thin metal film of SPR structures, directly in the Kretschmann configuration.

## 1. Introduction

The surface plasmon resonance (SPR) effect is the base of a mature technology that has a number of applications in physics [1,2,3], chemistry [4], biology [5], and other fields. The SPR phenomenon, which is related to a large variety of physical/chemical processes at interfaces, is based on the interaction of light with free electrons at a metal-dielectric interface [6]. The collective oscillations of free electrons, called surface plasmons (SPs), can be optically excited at that interface by the attenuated total reflection (ATR) if the resonance condition is fulfilled [1,2,3].

The most efficient way for generating the SPs provides the Kretschmann configuration [1]. A prism of high refractive index is coated on its base with a thin metal film and the SPs are excited in the metal film by the ATR mechanism. The field of SPs decays exponentially beneath and above the boundary and matching of the resonance condition, which is extremely sensitive to changes in the refractive index of the surrounding medium, is accompanied by a drop of the power carried by reflected light wave. Consequently, the SPR phenomenon is manifested by changes in the intensity [4], phase [7], resonant angle [8] or the resonant wavelength [9] of the reflected light wave. In the Kretschmann configuration, the SPR phenomenon can be resolved in an angular or spectral domain (angular or wavelength interrogation). Considering the angular interrogation [10,11,12], a monochromatic beam is used and a sharp minimum (dip) is observed in the angular spectrum. Similarly, in the wavelength interrogation [13,14,15], a dip is observed in the reflection spectrum.

A major problem with the theoretical confirmation of a measured response of an SPR sensor is the characterization of a thin metal film in the sensor. As an example, a previous study of the response of the SPR sensor in the spectral domain [16], including also the phase response, confirmed a very good agreement between the experiment and theory when a desirable model of dielectric function of a thin gold film was adopted. The comparison was performed for one angle of incidence (approximately $60^{\circ }$) only, but when we extend the measurement to other angles, the agreement fails. Similarly, the response to a known analyte differs from theory [17]. Consequently, a technique to characterize a thin metal film needs to be applied. Generally, a lot of SPR-based techniques are available to determine the geometrical and optical constants of thin metal films [18,19,20]. For their dispersion characterization, the spectral techniques such ellipsometry [21,22,23], reflectometry [24], and SPR-based reflectometry [25,26] are possible. We prefer to measure the dielectric function by a simple and cost-effective method compared to standard approaches like spectral ellipsometry, and, in addition, our motivation is application of the same configuration we use in sensing.

In this paper, we show a new approach in measuring the dielectric function of a thin metal film of an SPR structure. The method utilizes the response of the structure to air, represented by the spectral dependence of the ratio of the reflectances of *p*- and *s*-polarized waves, measured in the Kretschmann configuration at different angles of incidence. For the SPR structure comprising an SF10 glass prism and a gold coated SF10 slide with an adhesion film of chromium, we show that by processing these dependences near the resonance wavelength and using the dispersion characteristics of a metal film according to a proposed physical model, the real and imaginary parts of the dielectric function of the gold film can be determined. The parameters of the dielectric function of the thin gold film according to the Drude–Lorentz model with two additional Lorentzian terms are obtained by a least squares method for such a thickness minimizing the difference between the measured and theoretical dependence of the resonance wavelength on the angle of incidence. In addition, the results show that the gold film is composed of homogenous and rough layers.

## 2. Experimental Method

**Figure 1:**
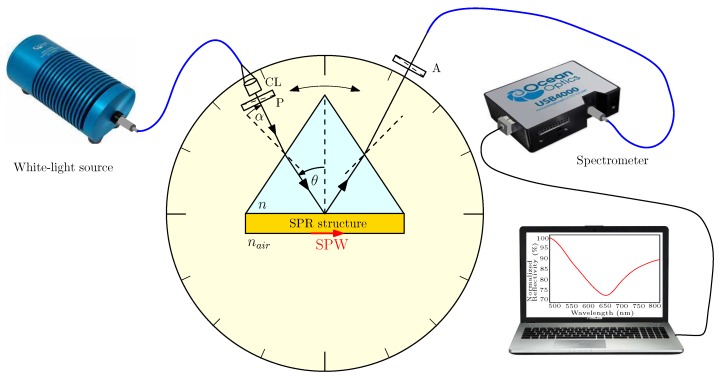
Experimental set-up: an SPR structure in the Kretschmann configuration; collimating lens (CL), polarizer (P), analyser (A).

In order to determine the dielectric function of a thin metal film, the wavelength dependence of the reflectance ratio $R_{p}\left(\lambda \right)/R_{s}\left(\lambda \right)$ of *p*- and *s*-polarized components for an incident light beam subjected to the SPR phenomenon at different angles of the incidence needs to be measured. The results are attained in an experimental set-up shown in Figure 1, which contains a white-light source (halogen lamp HL-2000, Ocean Optics, Dunedin, USA) with launching optics, collimating lens CL and an input optical fibre. The collimated beam of 1 mm diameter passes through linear polarizer P (LPVIS050, Thorlabs, Newton, USA) oriented $45^{\circ }$ with respect to the plane of incidence so that both *p*- and *s*-polarized components are generated. These polarized components undergo, owing to reflection from an SPR structure consisting of a high refractive index glass slide with a thin metal film attached to a glass prism by a thin film of index-matching fluid, the amplitude and phase changes that are related to the complex reflection coefficients

(1) $$r_{p,s}\left(\lambda \right)=\sqrt{R_{p,s}\left(\lambda \right)}\exp\left[i\delta_{p,s}\left(\lambda \right)\right],$$

where $R_{p,s}\left(\lambda \right)$ and $\delta_{p,s}\left(\lambda \right)$ are the wavelength-dependent reflectances and phase changes on reflection for both polarizations.

The reflected light passes through linear analyser A (LPVIS050, Thorlabs) oriented $0^{\circ }$ or $90^{\circ }$ with respect to the plane of incidence so that *p*- or *s*-polarized components are resolved. Because the ratio Rp/Rs is very sensitive to the polarizer/analyser accuracy, we verified that the output signal is zero when they are crossed and oriented $45^{\circ }$ with respect to the plane of incidence. The light is launched directly into a read optical fibre (M14L02, Thorlabs) of a spectrometer (USB4000, Ocean Optics), which is connected to a computer. In this case, no optical objective is used contrary to our previous experimental arrangement [14,16] so that the deterioration of the response near the resonance wavelength due to misalignment with respect to the output beam axis is minimized. To minimize the effect of an analyte on the measured dielectric function of a metal film, the SPR phenomenon is considered for air when a desirable angle of incidence is adjusted and the phase matching conditions are fulfilled.

This polarimetric method is based on measuring the ratio $R_{p}\left(\lambda \right)/R_{s}\left(\lambda \right)$ of the reflectances of *p*- and *s*-polarized waves at different angles of incidence. If the angle between the incident beam and the normal to the prism face (see Figure 1) is denoted as α, the angle of incidence θ on the base of the equilateral prism is

(2) $$\theta =60^{\circ }+\sin^{-1}\left[n\left(\lambda_{r}\right)\sin\alpha /n_{air}\left(\lambda_{r}\right)\right],$$

where $n(\lambda_{r})$ and $n_{air}\left(\lambda_{r}\right)$ are the refractive indices of the prism glass and air, respectively, at the resonant wavelength $\lambda_{r}$.

The SPR structure under study is represented by an SF10 glass slide substrate with an adhesion Cr film on which Au film was deposited. The corresponding thicknesses are $t_{1}$ = 2 nm and $t_{2}$ = 44.8 nm as specified by producer (Accurion, Goettingen, Germany). The SF10 glass slide with the deposited films was attached to an equilateral prism (SF10 glass) by a thin film of index-matching fluid (Cargille, Cedar Grove, USA, $n_{D}$ = 1.730).

**Figure 2:**
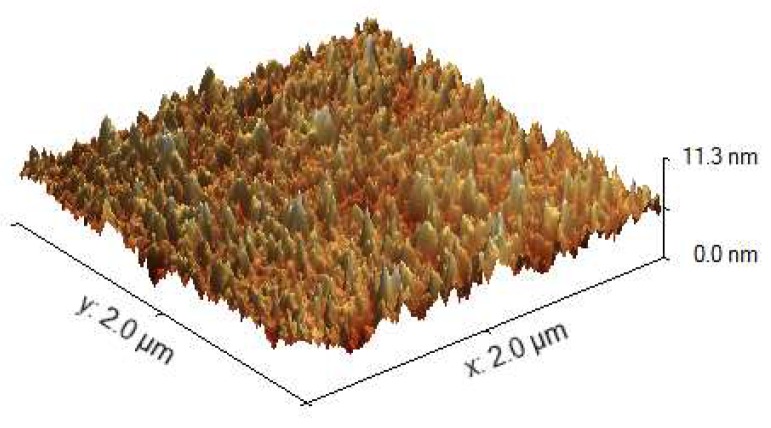
AFM image of a thin gold film on an SF10 glass substrate.

The surface morphology of a gold film on SF10 glass substrate was inspected by atomic force microscopy (AFM) using NTEGRA Prima (NT-MDT, Co., Moscow, Russia) in the semi-contact mode with a scanning area of 10 × 10 μm. An example of the result is shown in Figure 2 and it indicates that the surface roughness is nearly uniform in this area. By processing the obtained surface profile, the height distribution function and the RMS-roughness were determined [27], which provided the pseudolayer thickness $t_{3}$ = 2.2 nm and the volume fraction of the gold in it *q* = 0.5.

## 3. Processing Procedure

Our procedure is based on processing the spectral dependence of the ratio of the reflectances of *p*- and *s*-polarized waves measured in the Kretschmann configuration at different angles of incidence for air. In particular, these dependences are processed in the vicinity of a dip (near the resonance wavelength) using the dielectric function of a metal film according to a proposed physical model. The resultant dielectric function is determined by such a thickness provided the least squares difference between the measured and theoretical dependence of the resonance wavelength on the the angle of incidence.

### 3.1. SPR Structure—Reflection Coefficients

**Figure 3:**
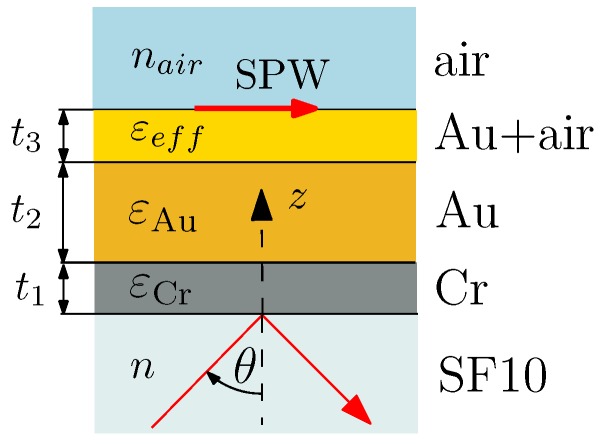
An SPR structure under study.

To proceed in processing procedure, the model of a five-layer system is adopted. The schematic drawing of an SPR structure is depicted in Figure 3, and it consists of the plate made of SF10 glass covered by a thin adhesion film of chromium between the plate material and gold film. The roughness of the gold surface is represented by the effective medium layer (pseudolayer), whose outer boundary is in contact with air.

In order to keep clear insight into the physics of the SPR structure, the physical parameters of all the films are included in the numerical processing based on the thin film system. The optical wave incident from the glass prism can be coupled into the surface plasmon wave (SPW) if the tangential component of incident wave matches the wave vector of the SPW.

The complex reflection coefficient of a five-layer structure is given by [28]

(3) $$r_{p,s(12345)}\left(\lambda \right)=\frac{r_{p,s(1,2)}\left(\lambda \right)+r_{p,s(2345)}\left(\lambda \right)\exp\left[i2k_{z1}\left(\lambda \right)t_{1}\right]}{1+r_{p,s(1,2)}\left(\lambda \right)r_{p,s(2345)}\left(\lambda \right)\exp\left[i2k_{z1}\left(\lambda \right)t_{1}\right]},$$

where

(4) $$r_{p,s(2345)}\left(\lambda \right)=\frac{r_{p,s(2,3)}\left(\lambda \right)+r_{p,s(345)}\left(\lambda \right)\exp\left[i2k_{z2}\left(\lambda \right)t_{2}\right]}{1+r_{p,s(2,3)}\left(\lambda \right)r_{p,s(345)}\left(\lambda \right)\exp\left[i2k_{z2}\left(\lambda \right)t_{2}\right]},$$

and

(5) $$r_{p,s(345)}\left(\lambda \right)=\frac{r_{p,s(3,4)}\left(\lambda \right)+r_{p,s(4,5)}\left(\lambda \right)\exp\left[i2k_{z3}\left(\lambda \right)t_{3}\right]}{1+r_{p,s(3,4)}\left(\lambda \right)r_{p,s(4,5)}\left(\lambda \right)\exp\left[i2k_{z3}\left(\lambda \right)t_{3}\right]}.$$

Here, $r_{p,s(j,k)}$ are the complex reflection coefficients of *p*- and *s*-polarized components at each interface. They are given by Fresnel formulae

(6) $$r_{p(j,k)}\left(\lambda \right)=\frac{{\tilde{n}}_{k}^{2}\left(\lambda \right)k_{zj}\left(\lambda \right)-{\tilde{n}}_{j}^{2}\left(\lambda \right)k_{zk}\left(\lambda \right)}{{\tilde{n}}_{k}^{2}\left(\lambda \right)k_{zj}\left(\lambda \right)+{\tilde{n}}_{j}^{2}\left(\lambda \right)k_{zk}\left(\lambda \right)},$$

and

(7) $$r_{s(j,k)}\left(\lambda \right)=\frac{k_{zj}\left(\lambda \right)-k_{zk}\left(\lambda \right)}{k_{zj}\left(\lambda \right)+k_{zk}\left(\lambda \right)},$$

where ${\tilde{n}}_{i}\left(\lambda \right)$ is the complex refractive index of the *i*-th layer, and $k_{zi}\left(\lambda \right)$ represents the normal wave vector component (along *z* axis) in it. An alternative way is to use a matrix formalism [29].

### 3.2. Material Characterization

Because the SPR phenomenon is very sensitive to material characterization of individual films and is studied in the spectral domain, the dispersion properties of all involved materials has to be described precisely. The dispersion of air and the change of refractive index of a prism with the temperature are also considered in the computation of the spectral responses.

#### 3.2.1. Air

The dispersion properties of air at a temperature of 15 ${}^{\circ }$C and a pressure of 101.3 kPa are described by a two-term Sellmeier-like formula

(8) $$n_{air}\left(\lambda ,T_{0},p_{0}\right)=n_{0}+\frac{A_{1}\lambda^{2}}{B_{1}\lambda^{2}-1}+\frac{A_{2}\lambda^{2}}{B_{2}\lambda^{2}-1},$$

where $n_{0}$ = 1.000064328, λ is the wavelength in μm and the Sellmeier coefficients $A_{i}$ and $B_{i}$ are as follows [30]: $A_{1}=2.94981\times 10^{-2}\;{\mu m}^{-2}$, $A_{2}=2.554\times 10^{-4}\;{\mu m}^{-2}$, $B_{1}=146\;{\mu m}^{-2}$, $B_{2}=41\;{\mu m}^{2}$.

The refractive index of air at temperature *T* and pressure *p* can be expressed as [30]

(9) $$n_{air}\left(\lambda ,T,p\right)=1+\frac{\left[n_{air}\left(\lambda ,T_{0},p_{0}\right)-1\right]}{1+\alpha (T-T_{0})}\frac{p}{p_{0}},$$

where $\alpha =3.4785\times 10^{-3}$ K${}^{-1}$ is the thermal expansion coefficient of air.

#### 3.2.2. SF10 Glass

The dispersion properties of a coupling prism made of SF10 glass are described at the reference temperature $T_{0}$ by a three-term Sellmeier formula

(10) $$n^{2}\left(\lambda ,T_{0}\right)=1+\sum_{i=1}^{3}\frac{A_{i}\lambda^{2}}{\lambda^{2}-B_{i}},$$

where λ is the wavelength in μm and $A_{i}$ and $B_{i}$ are the Sellmeier coefficients. Their values for SF10 glass at a temperature of 20 ${}^{\circ }$C are [31]: $A_{1}=1.61625977$, $A_{2}=0.259229334$, $A_{3}=1.07762317$, $B_{1}=0.0127534559\;{\mu m}^{2}$, $B_{2}=0.0581983954\;{\mu m}^{2}$ and $B_{3}=116.60768\;{\mu m}^{2}$.

The change in the refractive index $n(\lambda ,T_{0})$ with the temperature difference $\Delta T=T-T_{0}$ can be expressed as [31]

(11) $$\Delta n\left(\lambda ,T\right)=\frac{n^{2}\left(\lambda ,T_{0}\right)-1}{2n(\lambda ,T_{0})}\left(D_{1}\Delta T+D_{2}\Delta T^{2}+D_{3}\Delta T^{3}+\frac{E_{1}\Delta T+E_{2}\Delta T^{2}}{\lambda^{2}-\lambda_{TK}^{2}}\right),$$

where λ is the wavelength in μm and $D_{i},E_{i}$ and $\lambda_{TK}$ are temperature dispersion constants. Their values for SF10 glass are [32]: $D_{1}=5.31\times 10^{-6}$ K${}^{-1}$, $D_{2}=1.59\times 10^{-8}$ K${}^{-2}$, $D_{3}=-4.07\times 10^{-11}$ K${}^{-3}$, $E_{1}=1.28\times 10^{-6}$ K${}^{-1}$, $E_{2}=1.32\times 10^{-9}$ K${}^{-2}$ and $\lambda_{TK}=0.27\;\mu m$. The refractive index at temperature *T* is then

(12) $$n\left(\lambda ,T\right)=n\left(\lambda ,T_{0}\right)+\Delta n\left(\lambda ,T\right).$$

#### 3.2.3. Adhesion Film

The chromium adhesion film (thickness $t_{1}$) between the prism material and gold is described by an analytical Kramers–Kronning consistent model based on the critical point analysis [33], and the complex dielectric function of chromium is given by a three-term formula

(13) $$\begin{array}{cc} \varepsilon_{\mathrm{Cr}}\left(\lambda \right) & =\varepsilon_{\infty }-\frac{1}{\lambda_{p}^{2}\left(1/\lambda^{2}+i/\gamma_{p}\lambda \right)}\\ + & \sum_{j=1}^{2}\frac{A_{j}}{\lambda_{j}}\left[\frac{e^{i\phi_{j}}}{\left(1/\lambda_{j}-1/\lambda -i/\gamma_{j}\right)}+\frac{e^{-i\phi_{j}}}{\left(1/\lambda_{j}+1/\lambda +i/\gamma_{j}\right)}\right]. \end{array}$$

The dispersion of Cr dielectric function, which is described by Equation (13) with parameters specified in Table 1, is with an accuracy suitable for model computation within the spectral range from 400 to 1000 nm.

#### 3.2.4. Gold Film

To describe the complex dielectric function of gold, different models can be adopted, including the well-known Drude–Lorentz model, a combination of Drude and critical points models [23,33,34] and the Drude–Lorentz model with two additional Lorentzian terms [35]. The accuracy of the models, which increases with the number of parameters used, is sufficient in the near-UV/visible region. To describe the complex dielectric function of gold in a wavelength range from 534 to 908 nm, we adopted the Drude–Lorentz model with two additional Lorentzian terms [34,35] (successfully used in our previous paper [16]). The complex dielectric function of gold is then expressed by following equation:

(14) $$\varepsilon_{\mathrm{Au}}\left(\lambda \right)=1-\frac{1}{\lambda_{p}^{2}\left(1/\lambda^{2}+i/\gamma_{p}\lambda \right)}-\sum_{j=1}^{2}\frac{A_{j}}{\lambda_{j}^{2}\left(1/\lambda^{2}-1/\lambda_{j}^{2}\right)+i\lambda_{j}^{2}/\gamma_{j}\lambda }.$$

The dispersion of Au dielectric function, which is described by Equation (14) with parameters specified in Table 2, is with an accuracy suitable for model computation within the spectral range from 300 to 1000 nm.

#### 3.2.5. Effective Medium

Taking into account the surface roughness of the gold film, the layer of effective medium parameters needs to be included. The dielectric function $\varepsilon_{eff}\left(\lambda \right)$ of an added pseudolayer of thickness $t_{3}$ (see Figure 3) comprising the gold and air is determined according to the Maxwell–Garnett theory as [27]

(15) $$\varepsilon_{eff}\left(\lambda \right)=\varepsilon_{\mathrm{Au}}\left(\lambda \right)\frac{\left[\left(3-2q\right)\varepsilon_{air}\left(\lambda \right)+2q\varepsilon_{\mathrm{Au}}\left(\lambda \right)\right]}{\left[q\varepsilon_{air}+\left(3-q\right)\right]\varepsilon_{\mathrm{Au}}\left(\lambda \right)]},$$

where *q* represents the volume fraction of the metal in the pseudolayer.

## 4. Experimental Results and Discussion

Measurements were performed for a sample with no history and the reflectance ratio $R_{p}\left(\lambda \right)/R_{s}\left(\lambda \right)$ was obtained for 35 angles of incidence for which the SPR phenomenon was resolved. The reference position is for angle of incidence $\alpha =0^{\circ }$ when the light beam is incident perpendicularly to the prism face. Using the rotary stage, to which a collimator was attached, the angle of incidence can be adjusted in a desirable range.

**Figure 4:**
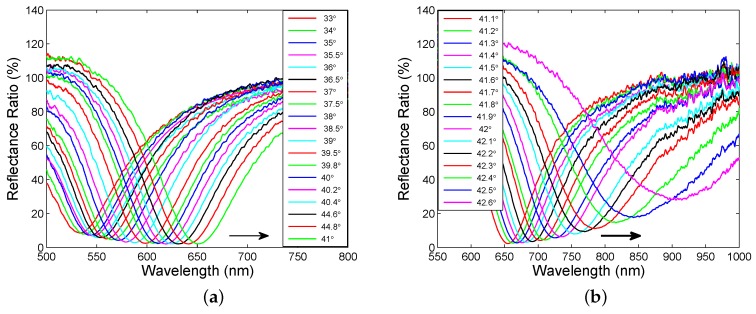
Measured reflectance ratio $R_{p}\left(\lambda \right)/R_{s}\left(\lambda \right)$ as a function of the wavelength for different angles of incidence α: $33^{\circ }$ to $41^{\circ }$ (**a**), $41.1^{\circ }$ to $42.6^{\circ }$ (**b**).

Figure 4a shows the measured reflectance ratio as a function of the wavelength for angle α ranging from $33^{\circ }$ to $41^{\circ }$, when steps were $1^{\circ }$, $0.5^{\circ }$ and $0.2^{\circ }$, respectively. The reflectance ratios have sufficiently pronounced dips with a width increasing with increasing angle of incidence. The wavelength of the dip, the resonance wavelength, shifts toward longer wavelengths and the reflectance ratio at the resonance wavelength is decreasing with increasing angle of incidence.

Similarly, in Figure 4b, the measured reflectance ratio as a function of the wavelength for angle of incidence ranging from $41.1^{\circ }$ to $42.6^{\circ }$ is shown with a step of $0.1^{\circ }$. Once again, the resonance wavelength shifts toward longer wavelengths with the increasing angle of incidence, but the change is greater. In addition, the reflectance spectra are broader and the reflectance ratio at the resonance wavelength increases with the increasing angle of incidence.

To determine the dielectric function of a gold film from the measured wavelength dependences of the reflectance ratio $R_{p}\left(\lambda \right)/R_{s}\left(\lambda \right)$, various approaches can be adopted. The most simple is based on a linear approximation of both the real and imaginary parts of the dielectric function in the vicinity of the resonance wavelength. This approach fails because of a nonlinear change of the imaginary part. The approximation can be improved by a quadratic function, but from the physical point of view such approximation needs to be justified. Hence, our approach is based on a modified Drude–Lorentz model given by Equation (14).

The processing procedure starts with a choice of thicknesses $t_{2}$ and $t_{3}$ of a gold film and pseudolayer, respectively, and, because of the thickness provided by the producer, the condition $t_{2}+t_{3}=$ 44.8 nm is applied. The starting thickness for pseudolayer is 2 nm and changes are with a step of 0.1 nm. Similarly, the starting volume fraction of the gold in the pseudolayer is q=0.5 and changes are with a step of 0.1. Then, the measured reflectance ratio spectra for different angles of incidence are processed to obtain the corresponding dielectric constants as a function of the resonance wavelength. Using the individual dielectric constants thus obtained in a whole measured wavelength range, they are fitted to a model function and the resonance wavelength as a function the angle of incidence is determined. The best fit is related to the minimization of the function

(16) $$\begin{array}{c} F\left(x\right)=\sqrt{\frac{1}{N}\sum_{i=1}^{N}{\left[\lambda_{ri}^{T}\left(x\right)-\lambda_{ri}^{E}\right]}^{2}}, \end{array}$$

where $\lambda_{ri}^{E}$ is the measured resonance wavelength, $\lambda_{ri}^{T}\left(x\right)$ is the theoretical resonance wavelength computed using the model and *x* is a set of model parameters ($t_{2}$, $t_{3}$, *q*, $\lambda_{p}$, $\gamma_{p}$, $A_{j}$, $\lambda_{j}$, $\gamma_{j}$), and *N* is the number of measurements.

**Figure 5:**
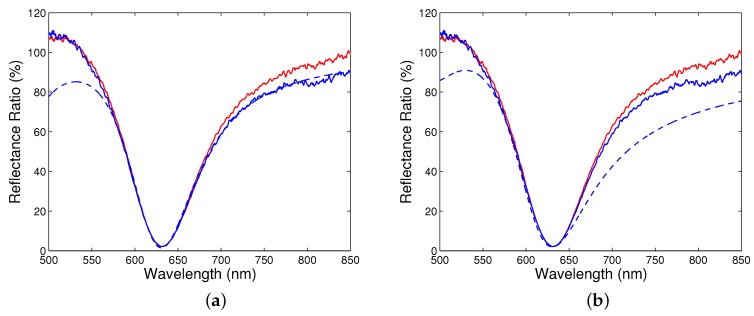
Measured (solid curves) reflectance ratio $R_{p}\left(\lambda \right)/R_{s}\left(\lambda \right)$ as a function of the wavelength for angle of incidence $\alpha =40.6^{\circ }$ together with the fitted one (**a**), and with the modelled one (**b**). Lower solid curve corresponds to a different loop diameter of a read optical fibre.

To illustrate the first step of processing, Figure 5a shows the result for the reflectance ratio $R_{p}\left(\lambda \right)/R_{s}\left(\lambda \right)$ measured at an angle of incidence of $40.6^{\circ }$ and at a temperature of 22 ${}^{\circ }$C. In fitting, the thicknesses of individual films were $t_{1}$ = 2 nm, $t_{2}$ = 42.8 nm and $t_{3}$ = 2.0 nm, respectively, and the volume fraction of the gold in the pseudolayer was *q* = 0.5. These parameters are the result of the processing procedure in the thickness ranges for gold film and pseudolayer, and with the changes of the volume fraction of gold as specified above. The last two parameters are in very good agreement with the results of the AFM inspection. Fitting was limited to a 100 nm wavelength range around a resonance wavelength of 631.52 nm near which the response is not dependent on the loop diameter of a read optical fibre (see Figure 5a). The starting parameters $\lambda_{p}$, $\gamma_{p}$, $A_{j}$, $\lambda_{j}$, $\gamma_{j}$ in the modified Drude–Lorentz model (14) were taken from Table 2.

The fitting procedure was applied to the remaining wavelength dependences of the reflectance ratio shown in Figure 4a,b. The values of the corresponding real and imaginary parts of the dielectric function were then used to determine the resultant function. Figure 5b shows a comparison of the reflectance ratio $R_{p}\left(\lambda \right)/R_{s}\left(\lambda \right)$ measured for angle of incidence $\alpha =40.6^{\circ }$ with the modelled one. It is clearly seen that the position of the dip and the curves in a short wavelength range from the resonance wavelength agree very well, but the difference between the curves in a long wavelength range is greater. The difference between the measured and theoretical dependences can be attributed to the effect of the read optical fibre whose response is dependent on the fibre loop diameter (see Figure 5b). In addition, the non-ideal collimated beam can affect the spectral wings of the surface plasmon reflectance ratio [36].

**Figure 6:**
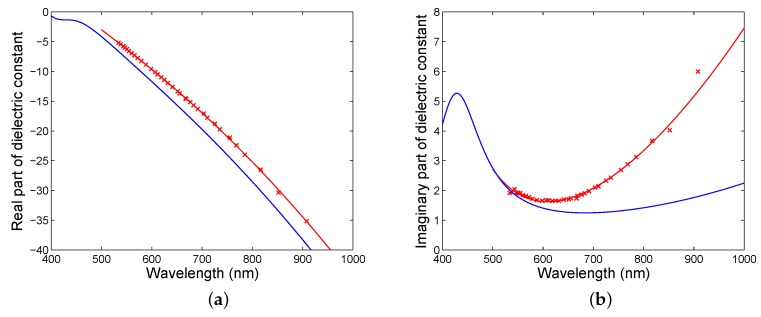
Dielectric function of the gold film (crosses) with a fit according to a modified Drude–Lorentz model: a real part (**a**), an imaginary part (**b**).

Next, Figure 6a shows the real part of the dielectric function thus obtained together with a fit according to the model function given by Equation (14). In the same figure is also shown the real part of the dielectric function corresponding to a model with parameters from Table 2. Similarly, in Figure 6b, the imaginary part of the dielectric function is shown together with a fit, and the reference function is also shown. The values of the real and imaginary permittivites corresponding to a resonance wavelength of 631.52 nm are −11.964 and 1.651, respectively. Considering that the spectra are recorded with a wavelength uncertainty of 0.2 nm, the errors of the real and imaginary permittivities are ±0.014 and ±0.001, respectively. The error of the real permittivity increases with increasing wavelength, while the error of the imaginary permittivity is variable with wavelength. As an example, at a wavelength of 908.16 nm, the real and imaginary permittivities are −35.179 and 5.995, respectively, with errors ±0.020 and ±0.004, respectively.

**Figure 7:**
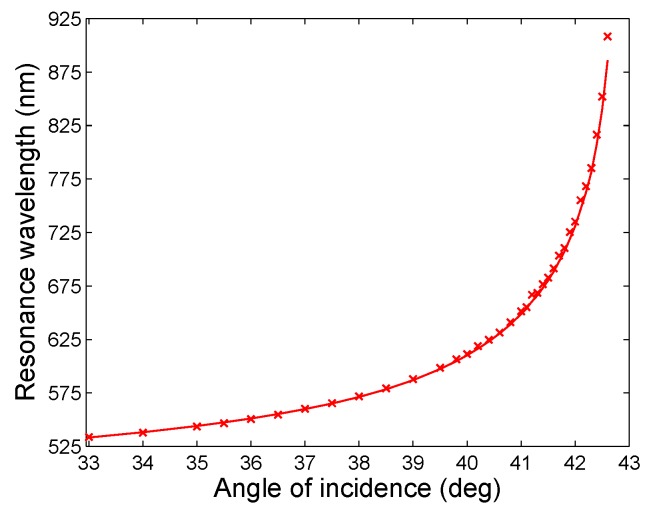
Measured (crosses) and modelled resonance wavelength as a function of the angle of incidence.

Both parts of the dielectric function, parameters of which are specified in Table 3, differ from a modified Drude–Lorentz model of Au with parameters from Table 2. They are responsible for very good agreement between the measured and theoretical response dependences. This can be demonstrated for the function of the resonance wavelength on the angle of incidence as depicted in Figure 7. The theoretical function corresponds to the best fit with the minimization of the function F(x) according to Equation (16), when the thicknesses of individual films are $t_{1}$ = 2 nm, $t_{2}$ = 42.8 nm and $t_{3}$ = 2.0 nm, respectively, and the volume fraction of the gold in the pseudolayer is *q* = 0.5. It is evident that the measured values agree very well with theoretical ones in a short wavelength range. In a long wavelength range, a slight difference is present which is caused by a greater error in measuring the resonance wavelength at greater angles of incidence. If we consider wavelengths 631.52 nm and 903.16 nm, respectively, and if the error in adjusted angle of incidence is $\pm 0.01^{\circ }$, the resonance wavelengths are with errors ±0.40 nm and ±5.36 nm, respectively, and the corresponding errors of the real permittivities are ±0.028 and ±0.536, respectively. Similarly, the errors of the imaginary permittivities are ±0.002 and ±0.107, respectively.

## 5. Conclusions

In this paper, a new approach in measuring the dielectric function of a thin metal film of an SPR structure is presented. The technique utilizes the response of the structure to air represented by the spectral dependence of the ratio of the reflectances of *p*- and *s*-polarized waves measured in the Kretschmann configuration at different angles of incidence. We show that by processing these dependences near the resonance wavelength and using the dispersion characteristics of a metal film according to a proposed physical model, the real and imaginary parts of the dielectric function of the thin metal film can be determined. The resultant dielectric function is obtained for such a thickness minimizing the difference between the measured and theoretical dependence of the resonance wavelength on the angle of incidence.

The feasibility of the technique has been demonstrated for an SPR structure comprising an SF10 glass prism and a gold coated SF10 slide with an adhesion film of chromium, when the Drude–Lorentz model with two additional Lorentzian terms for gold was adopted. In particular, the results show that the parameters of the gold film are affected by the surface roughness and, consequently, two particular thicknesses have to be included. The effective layer (psedolayer) thickness was in good agreement with the results obtained by the AFM technique.

To reach even better precision of measurement of the dielectric function in a long wavelength range, a finer adjustment of the angle of incidence needs to be attained. In addition, the use of the technique can be extended to the SPR phase differences [16] measured at different angles of incidence. Contrary to the reflectance ratios, these are not so sensitive to the response function of a read fibre of a spectrometer. Finally, the technique is of a particular importance in measuring the thickness and the dielectric function of thin metal films of SPR structures, directly in the Kretschmann configuration. 

## Figures and Tables

**Table 1:** Parameters of dielectric function of Cr [16,33].

Drude Term Parameter	Value	Oscillator 1 Parameter	Value	Oscillator 2 Parameter	Value
$\varepsilon_{\infty }$	1.1297	$A_{1}$	33.086	$A_{2}$	1.659
$\lambda_{p}$ (nm)	213.67	$\lambda_{1}$ (nm)	1082.3	$\lambda_{2}$ (nm)	496.5
$\gamma_{p}$ (nm)	4849.8	$\gamma_{1}$ (nm)	1153.2	$\gamma_{2}$ (nm)	2559.7
-	-	$\phi_{1}$ (rad)	−0.25722	$\phi_{2}$ (rad)	0.83533

**Table 2:** Parameters of dielectric function of Au [16,35].

Drude Term Parameter	Value	Oscillator 1 Parameter	Value	Oscillator 2 Parameter	Value
$\varepsilon_{\infty }$	1	$A_{1}$	3.613	$A_{2}$	1.423
$\lambda_{p}$ (nm)	133.85	$\lambda_{1}$ (nm)	309.11	$\lambda_{2}$ (nm)	424.06
$\gamma_{p}$ (nm)	27,851.5	$\gamma_{1}$ (nm)	2591.3	$\gamma_{2}$ (nm)	1515.2

**Table 3:** Parameters of dielectric function of Au retrieved from the experiment.

Drude Term Parameter	Value	Oscillator 1 Parameter	Value	Oscillator 2 Parameter	Value
$\varepsilon_{\infty }$	1	$A_{1}$	8.88	$A_{2}$	1.70
$\lambda_{p}$ (nm)	130.77	$\lambda_{1}$ (nm)	255.5	$\lambda_{2}$ (nm)	660.67
$\gamma_{p}$ (nm)	6608.3	$\gamma_{1}$ (nm)	−29.73	$\gamma_{2}$ (nm)	−819.68
